# *Anopheles* aquatic development kinetic and adults’ longevity through different seasons in laboratory and semi-field conditions in Burkina Faso

**DOI:** 10.1186/s13071-024-06260-2

**Published:** 2024-04-08

**Authors:** Nicaise D. C. Djègbè, Dari F. Da, Bernard M. Somé, Lawata Inès G. Paré, Fatoumata Cissé, Wadaka Mamai, Karine Mouline, Simon P. Sawadogo, Joseph D. Challenger, Thomas S. Churcher, Roch K. Dabiré

**Affiliations:** 1 Institut de Recherche en Sciences de la Santé, Direction Régionale, 399 avenue de la liberte, 01 BP 545 Bobo-Dioulasso 01, Burkina Faso; 2 Université Nazi Boni, Bobo-Dioulasso, Burkina Faso; 3 Institut de Recherche Agricole pour le Développement (IRAD), PO. Box 2123, Yaoundé, Cameroon; 4 MIVEGEC, Montpellier University, IRD, CNRS, Montpellier, France; 5 Medical Research Council Centre for Global Infections Disease Analysis, Department of Infectious Disease Epidemiology, Imperial College London, London, UK

**Keywords:** Temperature, Relative humidity, *Anopheles gambiae*, Dry season, Rainy season

## Abstract

**Background:**

*Anopheles* mosquitoes are ectothermic and involved in numerous pathogen transmissions. Their life history traits are influenced by several environmental factors such as temperature, relative humidity and photoperiodicity. Despite extensive investigations of these environmental conditions on vector population ecology, their impact on the different life stages of *Anopheles* at different seasons in the year remains poorly explored. This study reports the potential impact of these abiotic factors on the immature and adult stages of *Anopheles gambiae* sensu lato during different seasons.

**Methods:**

Environmental conditions were simulated in the laboratory using incubators to mimic the environmental conditions of two important periods of the year in Burkina Faso: the peak of rainy season (August) and the onset of dry season (December). Eggs from wild *An. coluzzii* and *An. gambiae* s.l. were reared separately under each environmental condition. For *Anopheles coluzzii* or *An. gambiae* s.l., eggs were equally divided into two groups assigned to the two experimental conditions. Four replicates were carried out for this experiment. Then, egg hatching rate, pupation rate, larval development time, larva-to-pupae development time, adult emergence dynamics and longevity of *Anopheles* were evaluated. Also, pupae-to-adult development time from wild L3 and L4 *Anopheles* larvae was estimated under semi-field conditions in December.

**Results:**

A better egg hatching rate was recorded overall with conditions mimicking the onset of the dry season compared to the peak of the rainy season. Larval development time and longevity of *An. gambiae* s.l. female were significantly longer at the onset of the dry season compared than at the peak of the rainy season. Adult emergence was spread over 48 and 96 h at the peak of the rainy season and onset of dry season conditions respectively. This 96h duration in the controlled conditions of December was also observed in the semi-field conditions in December.

**Conclusions:**

The impact of temperature and relative humidity on immature stages and longevity of *An. gambiae* s.l. adult females differed under both conditions. These findings contribute to a better understanding of vector population dynamics throughout different seasons of the year and may facilitate tailoring of control strategies.

**Graphical Abstract:**

**Figure:**
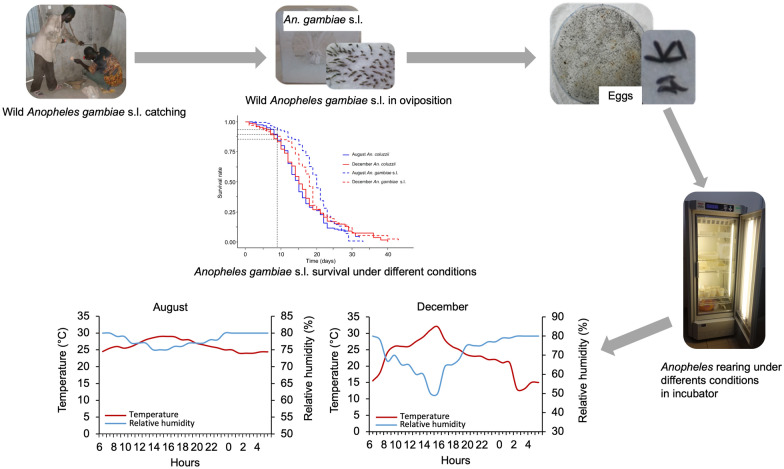

**Supplementary Information:**

The online version contains supplementary material available at 10.1186/s13071-024-06260-2.

## Background

Most disease vectors are ectothermic insects [1, 2] whose life history traits are modulated by ambient environmental conditions such as temperature, humidity and rainfall. In addition to influencing the vector itself, these environmental conditions can also affect the development of the microorganisms it harbors, impacting their transmission or extinction within the vector. *Plasmodium*, the malaria parasite, transmitted by *Anopheles* females, requires approximately 9–14 days to complete its sporogonic development within the mosquito vector [3, 4]. This period, called the extrinsic incubation period (EIP) [5], requires sufficient *Anopheles* longevity to achieve the transmission of *Plasmodium*. Before reaching the adult stage, the aquatic stage development of *Anopheles* occurs in breeding sites. Both the adult and immature mosquitoes are exposed to variable ecological conditions.

In malaria control efforts, several tools, either existing or under development, target different stages of vector development. For example, use of long-lasting insecticide-treated nets (LLINs) [6, 7], indoor residual spraying (IRS) [6, 8] and ivermectin [9] focuses on killing adult mosquitoes or reducing their longevity, thus reducing the probability of the completion of parasite’ sporogonic development. Control strategies for aquatic stages are mainly based on application of chemical insecticides [10] or biolarvicides [11–16] to the larval breeding sites and to a lesser extent on environmental management [16]. The priority of disease control programs is not only to reduce the survival rate of adult mosquitoes but also to manage the immature stages [17]. Targeting different development stages of *Anopheles* mosquitoes for control strategies requires a deeper understanding of the impact of environmental factors on the different phases for successful implementation of interventions. Mosquito life history traits are naturally regulated by abiotic and biotic factors. It has been reported that abiotic factors such as temperature and relative humidity play an important role in mosquito survival [18], and a review by Agyekum et al. successfully summarized the influence of temperature and relative humidity on mosquito traits [19]. However, most studies addressing this question have mainly investigated the phenomenon in laboratory conditions (constant temperature and relative humidity), excluding the wide variations of these parameters which occur in the field. Naturally, the regular temperature fluctuations experienced by most insects are more likely to have different effects on their development than exposure to constant temperatures. In addition, since humidity can depend on temperature, the effects of the two factors are closely related and difficult to isolate in the insect's natural environment [20]. Although many studies have explored the effect of temperature and relative humidity on mosquito life traits, there is limited evidence regarding the combined effect of their variation at different periods of the year on aquatic and adult stages of *Anopheles*. This question is difficult to address in the field because of the lack of precision due to unsuitable conduct of activities and limited temperature ranges where field experiments are undertaken [21]. Thus, Service [22] found that field conditions cannot be easily imitated and controlled laboratory studies are the best way to assess the combined effect of all the different factors on insects.

In the sub-Saharan Africa context, where resources allocated to malaria control are usually limited, the impact of environmental conditions at different times of the year on the aquatic stages of *Anopheles* (by slowing their development or reducing their density) could be crucial in guiding the methods used to control mosquitoes at these stages. For example, biolarvicides targeting *Anopheles* aquatic stages have a low persistence over time [16]. A period of the year when *Anopheles* larvae are developing slowly could be selected for multiple application of these biolarvicides, such as bacteria in the larval habitats, to reach large numbers. Additionally, the adult stages of *Anopheles* could also be targeted by examining the effect of environmental conditions at different times of the year on their longevity. An unfavorable period for *Anopheles* density or longevity could be used to intensify the use of vector control methods targeting these transmission parameters for greater impact.

This study aimed to investigate how temperature and relative humidity variation can affect mosquito development rate by mimicking two important seasons in western Burkina Faso: the peak of the rainy season (August) and onset of the dry season (December). To achieve our objective, we used two *Anopheles* groups, *An. coluzzii* and *An. gambiae* s.l., found in Bama and Soumousso respectively. These periods were chosen because mosquito abundance grows substantially at the peak of the rainy season (which occurs in August) and diminishes at the onset of the dry season (which occurs in December). Additionally, the peak of the rainy season is the period during which temperature fluctuations flatten and the ponds refill, while the onset of the dry season is characterized by important daily fluctuations in air temperature and a significant drop in relative humidity [23]. Also, the onset of the dry season is the transitional phase between the rainy season and the dry season, when insects such as mosquitoes undergo morphological and physiological changes to cope with the difficult period [24].

## Methods

### Selection of time period and environmental parameter simulation inside incubators

**Fig. 1:**
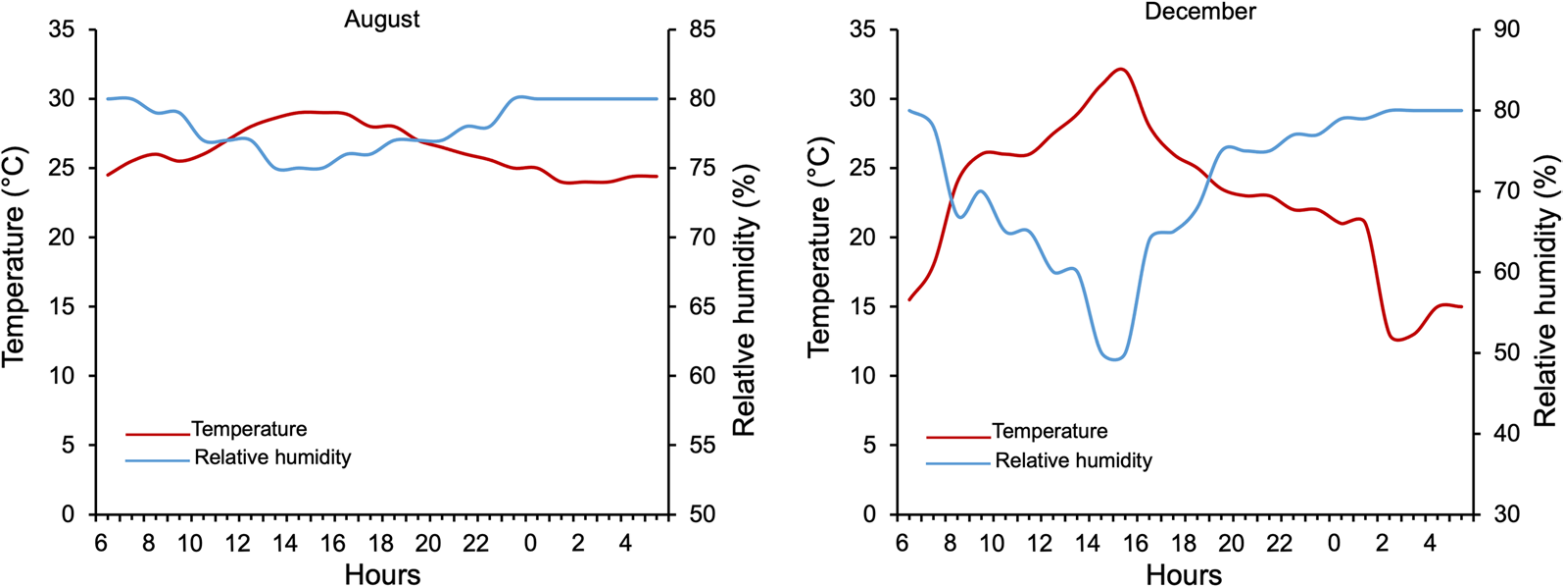
Hourly mean variation of temperature and relative humidity recorded during the months of August (peak of the rainy season) and December (onset of the dry season) in the village of Bama (western Burkina Faso). These data provide mean values from local records during a 4-year period (2009–2012). These values were reproduced in the incubators to simulate environmental conditions during the months of August (at the left) and December (at the right)

Based on the current weather parameters recorded in the village of Bama (Western Burkina Faso) during 4 consecutive years (2009–2012) using a local weather station (Vantage Pro2 weather monitoring station, Weatherlink; Davis Instruments, Hayward, CA, USA). The incubators were calibrated by adjusting the temperature and relative humidity (RH) to cyclically reproduce the environmental conditions in which the mosquitoes were reared. Two main seasons were simulated: the peak of the rainy season (August), corresponding to vector abundance combined with the highest transmission of *Plasmodium*, and the onset of the dry season (December) during which a relative decrease in the number of vectors was observed in the natural setting [25, 26]. In the IRSS laboratory, environmental conditions were simulated to mimic these two climatic periods inside two incubators (Sanyo MLR 315H, Sanyo Electric Co., Osaka, Japan). Thus, temperature and relative humidity (RH) cycles were programmed inside the climate chambers based on hourly recorded data in the village of Bama using a Vantage Pro2 weather monitoring station (Weatherlink; Davis Instruments, Hayward, CA, USA) for August and December [27]. In Bama village, these two parameters remain almost constant during the August period while important variations are observed between night and day in December (Fig. 1). For each period, hourly average temperature and RH were programmed to complete the daily weather cycle. We designed a 12-step cycle to be as close as possible to the natural daily climatic fluctuations in the climate chambers. A photoperiod of 12:12 h light/dark cycle was performed for all experiments because the minimal variations in photoperiod are observed in the tropics. About 1 h and 15 min day length variation is recorded between the solstices in Bobo-Dioulasso, Burkina-Faso, requiring the same photoperiod for the peak of the rainy season and onset of the dry season [23].

### Source of mosquitoes

The mosquitoes used for this study originate from F1 generation of wild *Anopheles* species (*An. coluzzii*, *An. gambiae* and *An. arabiensis*). F1 generation was obtained from wild blood-fed, semi-gravid and gravid *An. gambiae* s.l. collected during rainy season using a mouth aspirator in human dwellings in the rural localities of Bama and Soumousso. Both localities are located about 30 km and 50 km from Bobo-Dioulasso respectively. *Anopheles* mosquitoes from Bama are known to be essentially *An. coluzzii*, while in Soumousso, *An. coluzzii*, *An. gambiae* and *An. arabiensis* are found [28, 29]. All collected mosquitoes, morphologically identified as belonging to *An. gambiae* s.l. using the morphological keys of Giles and Coetzee (1987) [30], were kept in cages at the IRSS insectary (27 ± 2 ℃, 70 ± 5% relative humidity, 12:12 light/dark). They were supplied with 10% glucose solution ad libitum on cotton wool for about 3 days, the required time allowing egg development and the maximum number of females to lay eggs in Petri dishes lined with humid filter paper disks. After they laid eggs, wild-caught *An. gambiae* s.l. females were further identified to the species level using the SINE PCR [31].

### Mosquito rearing procedures in simulated environmental conditions

The collected eggs of *Anopheles* from different localities were equally divided into two groups for mosquito rearing in the two experimental conditions (August and December) inside incubators. For each experimental group, about 200 eggs were manually pooled under a stereomicroscope and transferred to plastic trays containing 1 l water that had been previously kept inside climatic chambers so that it was at an optimal temperature for each environmental condition. The emerged early-stage larvae were counted on the 3rd and 4th day after egg immersion. Subsequently, the larvae were transferred into other trays where they were reared using local spring water, which was changed every other day. Larvae were fed ad libitum with Tetramin^®^ Baby Fish Food (borehamwood, London, UK). Dead larvae were removed daily from each experimental group until the first pupae appeared. Pupae were counted daily and transferred into plastic cups, half-filled with spring water, and placed into cages for emergence into the climatic chambers. From this stage, the number of emerged mosquitoes was recorded every day. The non-emerged pupae after 24 h were placed in a new cage, taking care to remove the dead ones. This process was performed until all pupae emerged. Ten plastic trays were used per condition owing to five per locality. This experiment was conducted twice.

Different performance metrics across different transitions were recorded including egg hatching rate, larvae-to-pupae development time, pupae appearance dynamics, pupation rate, pupae-to-adult development time and adult longevity from *An. gambiae* s.l. The position of the trays was alternatively changed inside the climate chambers every 2 day in the morning to prevent any potential effect of the tray position. Furthermore, incubators were rotated between replicates to eliminate any relative effects.

### Estimating mosquito longevity in environmental simulated conditions

Adult *An. gambiae* s.l. mosquitoes collected from the experiments described above were used to estimate mosquito longevity in each experimental group. One day post-emergence, about 30 *Anopheles* females were sampled for each experimental group, kept in a plastic cup (V = 473 cm^3^) and maintained in incubator conditions with a sugar meal of 10% glucose ad libitum. Every morning, dead mosquitoes were recorded for each experimental group until all individuals were dead. Twenty (20) plastic cups containing approximately 30 mosquitoes were used for each experimental group. The same design was used for the experimental replicate. About 5% of the F1 generation from wild mosquitoes was molecularly analyzed to confirm the three species in the *Anopheles* population. Any random effect such as plastic cup position and climatic chambers was avoided by proceeding as described in the previous section.

### Measuring mosquito emergence in semi-field conditions in the December period

Based on our observations that the development time from pupae to adult *Anopheles* is very slow in December compared to August (similar to the literature reports) in incubators, it was interesting to better clarify this question through additional investigation in the semi-field environment to control some parameters. Therefore, we conducted similar experiments in the semi-field conditions focusing on the pupae-to-adult development time and adult dynamics emergence during the month of December. The assays were thus conducted in a malaria-sphere in which mosquitoes were exposed to all environmental factors including temperature and RH while keeping the *Anopheles* in the malaria-sphere, an enclosed environment. This facility is located in Bama village where the IRSS team conducted previous research focused on mosquito ecology [32]. In practice, an important quantity of *Anopheles* larvae (L3 and L4 stage) was collected from the breeding sites in Bama villages in December 2021. Then, they were kept in plastic trays in the malaria-sphere under the influence of environmental factors and fed ad libitum with Tetramin^®^ Baby Fish Food (Borehamwood, London, UK). During pupation, about 500 pupae were placed in plastic cups with spring water and kept in cages covered with a mosquito net. Every 24 h, the numbers of emerged adults and remaining pupae were counted; dead pupae were also recorded and removed. This experiment was replicated four times throughout the entire month of December.

### Statistical analyses

All data analyses were carried out using R software (version 4.0.2). Egg hatching, pupation and emergence rate respectively, defined as the proportion of eggs transformed into first instar (L1) larval stage, the proportion of larvae transformed into pupae and the proportion of pupae that successfully developed into adults, were calculated. Mean larval-to-pupal development time, defined as average time taken by larvae to transform into pupae, was also determined. Logistic regression by generalized mixed linear models (GLMM, binomial errors, logit link; *lme4* package) was used to test the effects of condition and *Anopheles* groups on the egg hatching, pupation and emergence rates. The full model of GLMM included conditions and *Anopheles* groups as fixed effects and replicates as random effect. Wilcoxon Mann-Whitney test (non-parametric test) was performed to compare mean larval-to-pupal development time between two different conditions. Kaplan-Meier survival was used to carry out *Anopheles* aquatic development dynamics. Only larvae transformed into pupae were included in this analysis. To determine the variation in daily survivorship of adult *Anopheles* kept in two conditions, Kaplan-Meier survival analysis was performed. A Cox regression (proportional hazard model) was used to assess the effects of condition and species on *Anopheles* survival.

## Results

### Effect of climatic conditions of August and December on eggs hatching

**Fig. 2:**
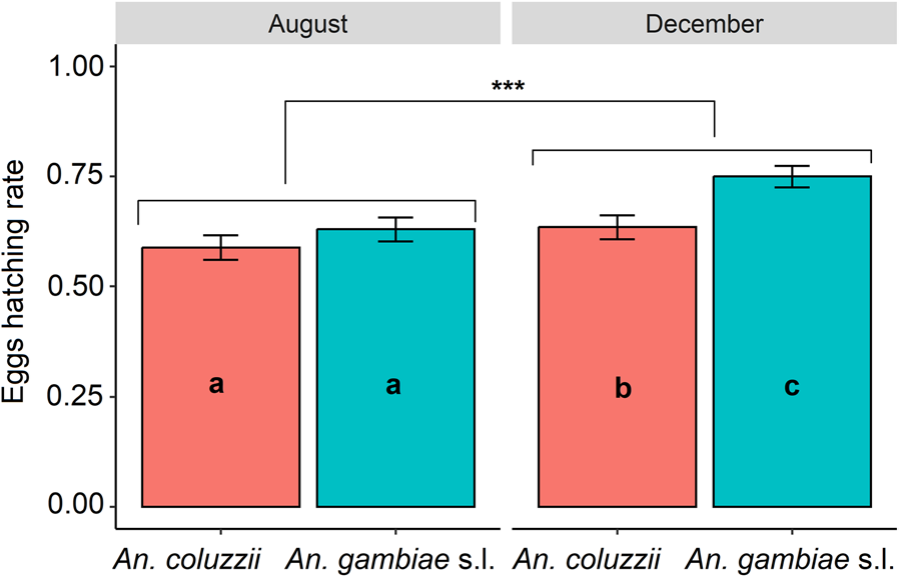
Eggs hatching rate of *An. coluzzii* and *An. gambiae* s.l. under August (peak of the rainy season) and December (onset of the dry season) conditions. Letters indicate groups that were significantly different. ***Significant difference between August and December

In total > 1000 wild adult *Anopheles* females were collected from Bama and 1260 from Soumousso, so the population likely contains a diverse genetic background for egg laying. All *Anopheles* females from Bama were *An. coluzzii*, in line with many previous works [28, 29], while three *Anopheles* species were identified for Soumousso females at various proportions: 51.6% *An. gambiae*, 38.2% *An. arabiensis* and 10.2% *An. coluzzii*. Overall, the environment parameter (temperature and RH variation) significantly affected mosquito egg development. When exposed to December conditions characterized by greater temperature and RH variation (mean temperature: 23 ± 1.11 ℃; mean RH: 71% ± 1.90%), the eggs had a high hatching rate compared to those under August conditions (mean temperature: 26 ± 0.35 ℃; mean RH: 78 ± 0.37%), independently of *Anopheles* group: 69% (95% CI 66–71) vs. 61% (95% CI 58–63) (likelihood ratio test: LRT, *X*^2^ = 18.36, *df* = 1, *P* < 0.001). The effect of environmental conditions on egg development was more important for both *An. coluzzii* and *An. gambiae* s.l. Indeed, both *An. coluzzii* and *An. gambiae* s.l. eggs were observed to have significantly higher development in December than in August conditions (Fig. 2).

### Kinetics of larval development to pupae

**Fig. 3:**
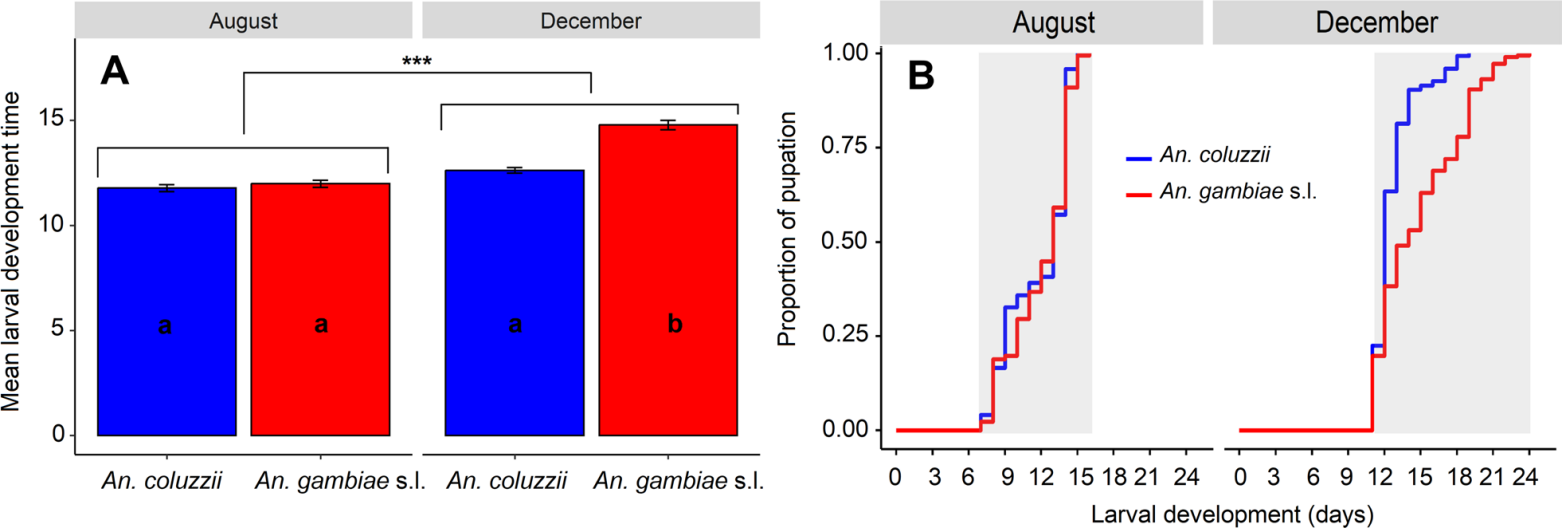
Larval development time (**A**) and temporal dynamics of the pupation (**B**) for the two simulated periods (August and December). Letters indicate groups that were significantly different, and day 0 corresponds to the day on which first instar larvae L1 were counted. ***Significant difference between August and December

*Anopheles* aquatic development dynamics performed using Kaplan-Meier survival analyses revealed significant differences between the two conditions (LRT, *X*^2^ = 52.8, *df* = 1, *P* < 0.001). After eggs hatched, the development of larvae required a longer time in December compared to August conditions (10 days vs. 6 days as minimal period) before the appearance of the first pupae. For *Anopheles* the mean duration of larval development from L1 stage to pupation was significantly shorter under August conditions than in December (11.87 ± 0.12 days vs. 13.82 ± 0.15 days; *W* = 73863; *P* < 0.001). In the *An. gambiae* s.l. group, a similar trend was seen with significantly faster development of larvae under August conditions than December conditions (11.98 ± 0.16 days vs. 14.77 ± 0.22 days; *W* = 15273; *P* < 0.001; Fig. 3A). From larva stage, the dynamics of the appearance of *Anopheles* pupae was faster under August than December conditions. Indeed, in August conditions, pupation started early with short duration (7–16 days post-egg hatching, shaded area) whereas the first pupae appeared later with long pupation time in December conditions (11–24 days post-eggs hatching, shaded area). Whatever the conditions, the pupae started appearing simultaneously for both *An. coluzzii* and *An. gambiae* s.l., with relatively shorter duration and less variability within the population of *An. coluzzii* (Fig. 3B).

### Pupation and the dynamics of adult emergence

**Fig. 4:**
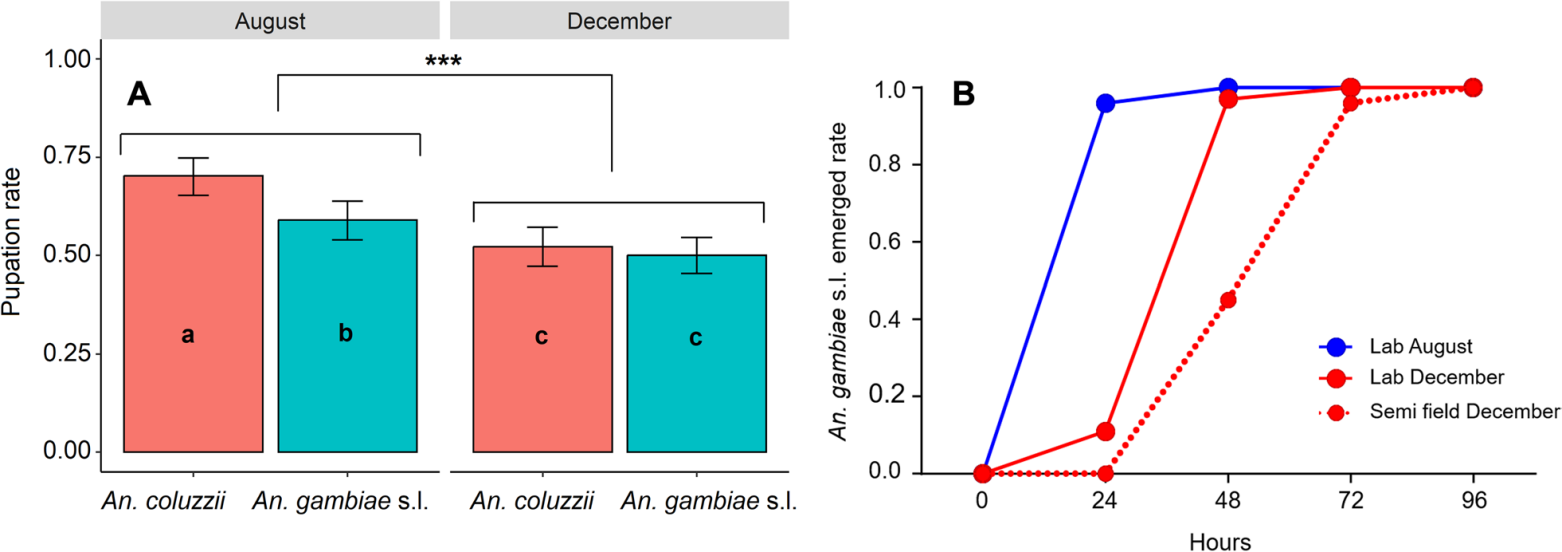
Larvae pupation rate (**A**) and emergence dynamics of *An. gambiae* s.l. (**B**) under the two simulated periods (August and December). Letters indicate groups that were significantly different. ***Significant difference between August and December

For the population of 1562 larvae resulting from *An. coluzzii* and *An. gambiae* s.l. egg hatching, temperatures and RH variation overall influenced the larval pupation. Conversely to egg hatching rate, the pupation rate was increased in *Anopheles* exposed to August conditions compared with their counterparts under December conditions, independently of *Anopheles* group [64% (95% CI 61–68) vs. 51% (95% CI 47–54); LRT, *X*^2^ = 28, *df* = 1, *P* < 0.001]. *Anopheles coluzzii* exhibited better pupation in August than in December conditions (LRT, *X*^2^ = 24.24, *df* = 1, *P* < 0.001). The same finding was found in *An. gambiae* s.l. between the two conditions (LRT, *X*^2^ = 6.33, *df* = 1, *P* = 0.01; Fig. 4A).

The time required by pupae metamorphosis to emerge into mosquito adult in each experimental group revealed two main periods for specimens reared in the laboratory independently of *Anopheles* group (Fig. 4B). While all pupae almost emerged in 24 h under August conditions, *Anopheles* adults have been recorded an additional 48 h later for those incubated in December conditions.

Like the dynamic of adult emergence in the laboratory, the same trend was observed for the wild *An. coluzzi* in semi-natural conditions in December. Indeed, 2093 pupae collected in the larval breeding sites in Bama and preserved in the malaria-sphere emerged as adults in 48 h and 72 h (Fig. 4B). For both laboratory-reared and wild mosquitoes, some pupae (< 2%) remained until 96 h before they emerged as adults under December conditions.

### Effect of different conditions on *An. gambiae *s.l. longevity

**Fig. 5:**
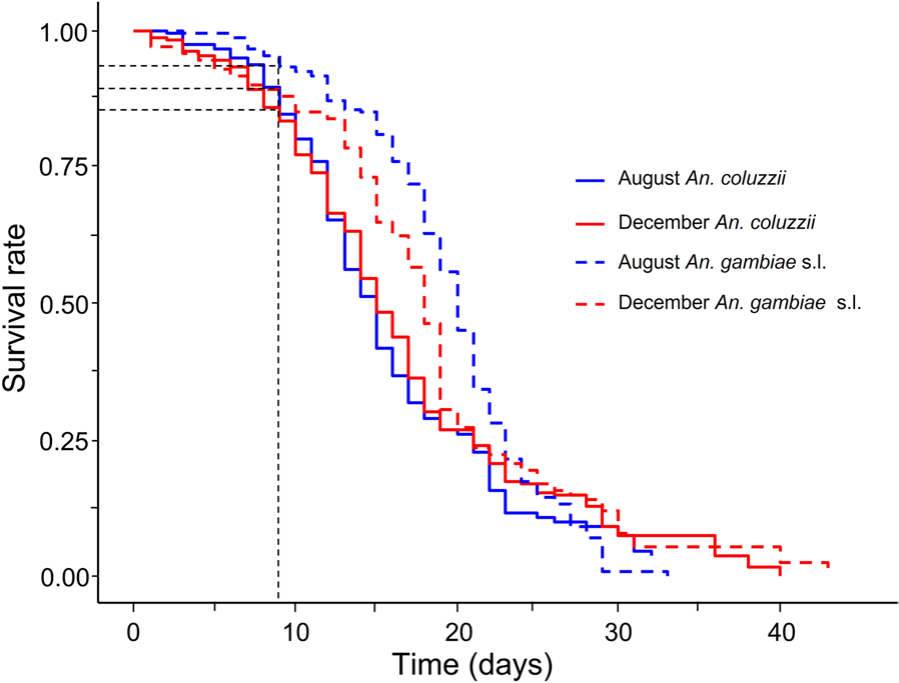
Effect of simulated temperature and relative humidity mimicking the environmental conditions of the periods of August (blue lines) and December (red lines) on *Anopheles* mosquitoes’ survival

As expected, *An. gambiae*, *An. arabiensis* and *An. coluzzii* were found in the F1 generation population of *An. gambiae* s.l. (47.4, 12.3 and 40.4% respectively). *Anopheles* females exposed to December conditions survived longer than those maintained in August conditions (LRT, *X*^2^ = 12.3, *df* = 1, *P* < 0.001; Fig. 5). Considered separately, *An. coluzzii* and *An. gambiae* s.l. showed significantly higher survival under December conditions than their counterparts under August conditions. In contrast to environmental dependence, mosquito survival was not associated with the *Anopheles* groups (LRT, *X*^2^ = 0.17, *df* = 1, *P* = 0.67). However, *An. coluzzii* and *An. gambiae* s.l. survived better in the right age range for *Plasmodium* transmission in the field (9–21 days). There was no significant interaction between *Anopheles* groups and environmental conditions (LRT, *X*^2^ = 0.95, *df* = 1, *P* = 0.33). When considering the *Anopheles* groups separately in the analyses, the environmental conditions affected the survival of *An. coluzzii* (LRT, *X*^2^ = 9.02, *df* = 1, *P* = 0.002) and *An. gambiae* s.l. (LRT, *X*^2^ = 11.2, *df* = 1, *P* < 0.001). Considering 9 days as the threshold age for *Anopheles* vectors to be infectious for *Plasmodium* transmission [3, 4], > 80% of mosquitoes achieved this age regardless of *Anopheles* group and environmental conditions.

## Discussion

This study explores the combined effect of temperature and relative humidity variations on *An. gambiae* s.l. development dynamics using two important periods (peak of the rainy season and onset of the dry season corresponding to August and December respectively). Some key life history traits of the *Anopheles* were assessed including egg hatching rate, dynamics of pupal appearance, larval development time, pupation rate, pupae-to-adult time and adult longevity. Overall, egg hatching was more successful under December than August conditions. These results suggest that December conditions characterized by higher fluctuating temperatures (15–32 ℃) and relative humidity (57.5–82.5%) provided a favorable environment for embryo development. A previous study showed that successful hatching of the eggs requires optimal development of the embryo [33]. The relatively high and almost constant temperatures and RH (24.5–28.5 ℃ and 76–80%) recorded in August led to an estimated reduction in the egg hatching rate of up to 45%. This suggests that the optimal development of the embryo is due to lower temperatures and RH. Also, for both *An. coluzzii* and *An. gambiae* s.l., the egg hatching rate was higher in December than in August conditions. Although this finding was obtained in the laboratory, it could be the same in the wild. This suggests rigorous environmental clean-up before December to eliminate vector oviposition sites.

In contrast, the development from larvae to pupae and the dynamics of pupal appearance were faster under August than December conditions regardless of *Anopheles* group. Similarly, pupation proportion was higher in August than in December. Specifically, under August conditions, first pupae appeared in 7 days with a high pupation rate, whereas in December conditions, first pupae occurred in 11 days with a low pupation rate, whatever *Anopheles* group. Additionally, pupae-to-adult time was spread over 48 h and 96 h under August and December conditions respectively. These observations suggest that August conditions with relatively higher temperatures and RH offer better larval development compared to December conditions. This could explain at least partially the higher anopheline population density observed in the natural environment during the August period, even though this density is more likely to be driven by the greater availability of breeding sites and nutrients favorable to larval development compared with December conditions. According to other studies, these temperature and relative humidity (RH) ranges (24.5–28.5 ℃ and 76–80%) observed under August conditions are optimal for the immature stage development to adult emergence [34, 35].

The mean larval-to-pupae development time reported under August conditions is similar to the values (11.5 days) found by Holstein since 1954 in the same locality [36]. In the same study, pupae-to-adult time was reported to spread over 48 h during the rainy season. The similarities in the finding between the two studies indicate that simulated environmental conditions in the laboratory can provide information from the field conditions. The findings revealed that pupae-to-adult time was spread over 4 days under controlled conditions of December programmed in the laboratory incubators. This same trend was found in the semi-field conditions in December, providing some support to the use of extrapolated laboratory results for the natural environment. Nevertheless, this verification was only done for one of the many experiments conducted in the laboratory, so care should be taken when extrapolating these results as they have not been validated in semi-field settings.

Overall, female *Anopheles* placed under December conditions (temperature from 15 to 32 ℃ and relative humidity from 57.5 to 82.5%) survived longer than those exposed to August conditions. This finding remained consistent when *An. coluzzii* and *An. gambiae* s.l. were considered separately in the analyses. December conditions are associated with cooler temperatures and lower relative humidity compared to August conditions, suggesting a relative tolerance of these mosquitoes to the abiotic factors present in December. A tolerance of the specimens to the December conditions would allow them better longevity. The high longevity observed in *Anopheles* female mosquitoes might also be attributed to an accumulation of lipid reserves that can allow mosquitoes to cope with acute drought conditions. It has been established that a significant accumulation of lipids leads to a hypertrophy of the fat body, and this mechanism increases survival in hibernating *Culex pipiens* females [37], which are holometabolous insects like *Anopheles* mosquitoes. The relationship between cooler temperatures and longevity could be explained in two ways. First, cooler temperatures could increase longevity by reducing the reaction rate of various metabolic processes that affect development and life history [38]. Second, lower temperatures could mitigate damage caused by metabolic by-products, such as reactive oxygen species [38]. Our findings related to mosquito longevity were in agreement with several previous studies. For instance, Hidalgo et al. [23] reported that the population of the complex *An*. *gambiae* reared under the onset of the dry season conditions (like December in Burkina Faso) lived longer than those reared under the peak of the rainy season conditions (August). According to these authors, the extended lifespan found at the onset of the dry season occurs because *Anopheles* enter a state of aestivation to overcome the long dry period. Mosquito longevity reported in this study may be underestimated because the specimens were not blood-fed. Indeed, it is reported that *An. gambiae* s.s. females fed with a combination of glucose and blood survived longer than those only fed with glucose [8]. We reported here that > 80% of mosquitoes survive beyond the threshold required for *Plasmodium* transmission, whatever the season. This value should be treated as illustrative as most mosquitoes will not be infected the day they emerge, and wild mosquitoes are likely to suffer substantially higher mortality than that observed in the laboratory. Nevertheless, this greater longevity could contribute to the high intensity of *Plasmodium* transmission recorded in the field from the peak of the rainy season to the onset of the dry season, as described by other authors [25]. This therefore stresses the need to reduce mosquito survival by using LLINs and IRS during these periods.

Although this study was not carried out in natural environments, it provided understanding of how *Anopheles* life history traits respond to two different environmental conditions of the year. Indeed, the development of the aquatic stages of *Anopheles* is considerably slowed down in December. Therefore, this period could be targeted by local control programs to multiply the application of biolarvicides in the few larval breeding sites still available at this time. Furthermore, *Anopheles* longevity is higher in December conditions. This should guide vector control programs in sensitizing the population for intensive use of adult mosquito control tools in December such as impregnated mosquito nets, which aim to reduce the longevity of *Anopheles*.

While our study has provided relevant findings, it would be interesting to envisage further studies considering a maximum number of dry and rainy season months.

## Conclusions

*Anopheles coluzzii* and *An. gambiae* s.l., from their immature to adult stages, were artificially exposed to two environmental conditions (temperature and relative humidity) simulating the peak of the rainy season (August) and onset of the dry season (December) in western Burkina Faso. Results revealed a high egg hatching rate, low dynamics of pupal appearance, slow larval development, slow pupae to adult time and high mosquito survival under onset of the dry season conditions compared to the peak of the rainy season conditions. These findings are useful for building a robust understanding of malaria vector populations dynamics at different times of the year to design tailored vector control strategies.

### Supplementary Information

**Additional file 1: **Dataset S1

## Data Availability

The data supporting the findings of the study is available in the Additional File 1.
